# Synthesis, Characterization, and Theoretical Modeling of 2D Molybdenum Boride (MBene) for All Solid-State Flexible Interdigitated Supercapacitor Application

**DOI:** 10.1021/acsomega.4c09727

**Published:** 2025-02-19

**Authors:** Parya Aghamohammadi, Fatma Karakaya Mert, Eda Taga Akgul, Nahid Aghabalapoor Keshtiban, Osman Cem Altıncı, Ali Gelir, Cem Sanga, Nadire Nayir, Hamide Aydın, Muslum Demir

**Affiliations:** † Department of Chemical Engineering, Osmaniye Korkut Ata University, Faculty of Engineering and Natural Sciences, 80000 Osmaniye, Turkey; ‡ Department of Physics Engineering, Istanbul Technical University, 34469 Istanbul, Turkey; § TUBITAK Marmara Research Center, Material Institute, 41470 Gebze, Turkey; ∥ Department of Chemical Engineering, Bogazici University, 34342 Istanbul, Turkey; ⊥ Paul-Drude-Institute for Solid State Electronics, Leibniz Institute within Forschungsverbund Berlin eV., Hausvogteiplatz 5-7, 10117 Berlin, Germany

## Abstract

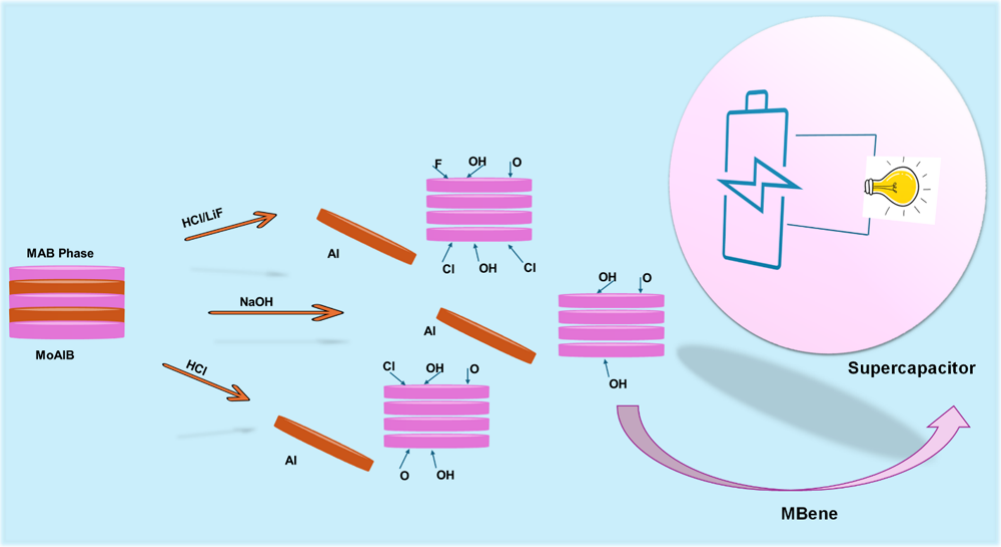

Recently, two-dimensional transition metal borides (2D MBenes) have demonstrated advanced physical, mechanical, and electrochemical performance; however, there are fewer studies in the literature. In particular, 2D molybdenum boride (2D MoB), created from molybdenum aluminum boride (MoAlB, MAB phase), is one of the important materials introduced due to its thermodynamically stable structure. Herein, we present the preparation of MoAlB and its chemical exfoliation by employing NaOH and LiF-HCl etching solutions to synthesize 2D MoB MBene nanostructures. It is found that the etching conditions significantly affect the selective extraction of the interlayer Al from the MAB phase in solution, forming 2D MoB MBene nanostructures. The resulting 2D MoB MBene illustrates sheet-like morphology, abundant redox active sites, and moderate textural features, which are critical for electrolyte accumulation and transfer. Powder X-ray Diffraction (PXRD) analysis confirmed the crystallinity and phase purity, while Scanning Electron Microscopy (SEM) and Transmission Electron Microscopy (TEM) revealed the sheet-like morphology and structural integrity. X-ray Photoelectron Spectroscopy (XPS) was employed to analyze the elemental composition and oxidation states, confirming the successful removal of Al and the formation of MoB MBene. Additionally, Brunauer–Emmett–Teller (BET) surface area analysis provided insights into the porous nature and surface area, which are crucial for electrolyte interactions. The all-solid-state flexible interdigitated supercapacitor (ISSC) device prepared using 10/MoAl_1–*x*_B electrode exhibited a good areal capacitance of 20.3 mF/cm^2^, outstanding cyclic capability with 92% capacitance retention after 1300 cycles, and remarkable flexibility. Last but not least, the Density Functional Theory (DFT) of the optimal MoB sample was constructed in terms of structural, conductivity, and thermodynamic stability perspectives, which is well-aligned with experimental results. Applying these 2D MoB MBene electrodes as all-solid-state flexible interdigitated electrodes opens a new avenue in supercapacitor technology.

## 1 Introduction

Supercapacitors have garnered substantial attention due to their outstanding high power density and long cycle life compared to alternative energy storage systems like batteries and fuel cells.^1−3^ The classification of these gadgets as electric double-layer capacitors (EDLCs) or pseudocapacitors (PCs) depends on the mechanism by which the electrode materials store charges during an electrochemical process.^4−6^ Although carbon-based EDLC materials exhibit strong cycling stability, they have low specific capacities.^7^ On the other hand, pseudocapacitive electrode materials such as oxides can overcome this limitation; however, they are prone to rapid capacity degradation during repeated charge and discharge cycles.^8−10^ To address this issue, among a wide variety of electrode materials, 2D materials have attracted attention due to their atomic-level thinness, theoretical high surface area, high charge carrier mobility, intriguing chemical reactivity, and exceptional mechanical durability.^11−13^

In particular, a novel category of transition metal boride layered nanomaterials is worth investigating because of their remarkable electrical conductivity, exceptional durability, and diverse surface chemistry.^13,14^ 2D multilayer MBenes are obtained through chemical etching and exfoliation of 3D MAB structures, which generally comprise a transition metal (M), elements from groups IIIA and IVA (A), and boron (B).^15^ The initial publication regarding MBenes can be traced back to 2017,^16^ during which they were regarded as resembling early transition metal carbides, nitrides, and carbonitrides (MXenes). However, MBenes have received comparatively less focus when compared to MXenes; however, have substantial potential in many fields thanks to the diverse valence states of boron atoms and their electron-deficient characteristics.^17^ Theoretical computations and initial experiments findings indicate a plethora of applications for 2D MBenes in magnetic devices,^16^ electrocatalysis,^18^ and batteries,^19^ making the exploration of these classes of compounds promising. In addition, layered materials maintain the mechanical and structural stability of electrodes during electrochemical reactions. Metal borides such as CoB and NiB have been synthesized using simple methods and investigated in asymmetric supercapacitors.^20−22^ However, the high cost of transition metals such as nickel and cobalt is encouraging the search for alternative electrode materials. Among the transition metals, metals such as molybdenum and tungsten are largely prominent. In addition to their unique physical and chemical properties, 2D MoB MBenes are inexpensive, highly electrically conductive, abundant, and environmentally friendly, making them particularly attractive to researchers for electronic applications.^23^ Various studies have demonstrated that the etching process can efficiently eliminate approximately 25% of the aluminum content in MoAlB. Nonetheless, current etching techniques usually involve the use of fluoride etching agents or high corrosion solutions at elevated temperatures, which are either not cost-effective or environmentally unfriendly and carry safety risks.^24−26^ One method to improve the electrochemical properties of MoAlB involves deintercalating aluminum from MoAlB with larger spacing. Molybdenum boride was synthesized, characterized by Vinoth et al.,^20^ and the electrochemical performance of the resulting layered MoB (LMB) electrode was evaluated with different aqueous electrolytes and found up to 445 and 130 F/g specific capacitance values in H_2_SO_4_ and Na_2_SO_4_, respectively. In another study, Wei et al.,^27^ introduced the synthesis of multilayered MoAl_1–*x*_B MBenes with different degrees of aluminum deintercalation, utilizing a gentle, fluoride-free method involving dilute alkali. The resulting MBene demonstrates terminal −OH groups and multilayered morphology as well as present advanced electrochemical performance of up to 1003.30 mF/cm^2^ areal capacitance. The present study is unique in terms of the systematic fabrication of MAB phase and MBene structures. Last but not least, the present experimental study is supported by DFT work which is not conducted in previous studies 2D MBenes are a very highly promising family of nanomaterials, despite being comparatively new and still under research. The MBenes seem quite similar to the MXene, but the carbon and/or nitrogen positions have been exchanged with the boron element. High surface area occurs as the Al element from the MAB molecule separates from the layers. In comparison to carbon, boron is lacking only one electron, which has received widespread interest because of its lack of electrons. The complexity of boron is due to its electronic structure: boron has three valence electrons, which makes it easy to show its tendency to build together with other boron atoms, thus forming complicated clusters and lattice structures.^28^

In the present study, we first fabricate the MoAlB phase by the solid-state pressure less sintering method. Then, MoAl_1–*x*_B compounds were synthesized with different Al deintercalations by removing Al from the MAB phase using traditional fluoride etchant LiF-HCl and dilute alkaline NaOH with room temperature or hydrothermal-assistant etching. The etching pathway and chemical reactions were explored and found that the LiF/HCl etching presented maximum Al removal from the MAB phase. We were in-depth investigated the electrochemical characteristics of electrode materials and concluded that the 10/MoAl_1–*x*_B electrode exhibited the highest ISSC performance. This study presents novel approaches for synthesizing MBenes-based electrodes, underscoring their noteworthy potential for utilization in ISSCs.

## 2 Experimental Section

### 2.1 Synthesis of Molybdenum Aluminum Boride

MoAlB was synthesized by the solid-state pressure less sintering method using a modification of a reported procedure.^29^ In brief, Mo powder (Sigma-Aldrich 99.99%), Al (Sigma-Aldrich 99.99%), and B powder (Alpha Aesar 98%) were mixed with a molar ratio of 2:1.5:2 for Mo:Al:B in a glovebox. The solid phase mixture was ball-milled for 2 h. The mixed powders were mechanically pressed under a pressure of 400 MPa for 2 min to form a disc. As-prepared 2 g discs were added to the crucible and thermally treated at 1500 °C with a ramp rate of 10 °C/min. The sample was held at this temperature for 10 h before cooling to 1000 °C at a rate of 1 °C/min and then cooled to room temperature at a rate of 3 °C/min.

### 2.2 Synthesis of 2D Molybdenum Boride

MoAlB was chemically exfoliated using two different etching solutions (NaOH and LiF-HCl) through a procedure similar to earlier reported literature^20,21,25,28^ with some modifications explained as follows. Briefly, in the first etching, 0.5 g of MoAlB powder was mixed with 40 mL of 25 wt % NaOH solution (pH of 13.76) and transferred to a 100 mL autoclave, and then the autoclave was heated at 160 °C for 24 h, after the hydrothermal process, a precipitate black color was obtained. The resulting product was washed with deionized water five times until pH ∼ 7. The black powder sample prepared by vacuum filtration was dried in the oven at 60 °C for 24 h and named 25HT/MoAl_1–*x*_B. In the second etching, 0.5 g of MoAlB powder was mixed with 40 mL 10 wt % NaOH solution (pH of 13.66) at 25 °C for 24 h and then the resulting product was washed with deionized water five times until pH ∼ 7 and vacuum filtrated. The as-prepared powders were dried in the oven at 60 °C for 24 h and named 10/MoAl_1–*x*_B. In the other etching, 0.5 g of MoAlB powder was etched with an aqueous solution of 1 g LiF in 6 M HCl (pH of 0.78) at 25 °C at room temperature for 24 h and the sample was the resulting product washed with deionized water several times until pH ∼ 7 and vacuum filtrated and dried in the oven at 60 °C for 24 h named LiF-HCl/MoAl_1–*x*_B.^1^ In the final etching, 0.25 g of MoAlB powder was mixed with 40 mL of 25 wt % NaOH solution (pH of 13.76) at 25 °C for 24 h. Then, the resulting product was washed with deionized water five times until pH ∼ 7 and vacuum filtrated. The as-prepared powders were dried in the oven at 60 °C for 24 h named 25/MoAl_1–*x*_B.^2^Table 1 depicts experimental steps in which nanostructures were obtained by chemical exfoliation of MoAlB using various etching agents.

**Table 1:** Experimental Conditions Applied for MoAl_1–*x*_B (MBene)

sample (MBene)	etching solution	MoAlB weight (g)	etching temperature	pH of the etching solution
25HT/MoAl_1–*x*_B	NaOH (40 mL, 25 wt %)	0.5	hydrothermal process, 160 °C	13.76
10/MoAl_1–*x*_B	NaOH (40 mL, 10 wt %)	0.5	26.5 °C	13.66
25/MoAl_1–*x*_B	NaOH (40 mL, 25 wt %)	0.25	26.5 °C	13.76
LiF-HCl/MoAl_1–*x*_B	1 g LiF, 40 mL 6 M HCl	0.5	26.5 °C	0.78

### 2.3 Physical Characterization

The structures of the obtained samples were made with X-ray powder diffraction analysis Rigaku SmartLab diffractometer at room temperature. Cu–Kα (λ = 1.5406 Å; 40 kV; 30 mA) radiation was chosen as the light source in the diffractometer. The morphologies of obtained MBene nanostructures were characterized with scanning electron microscopy (SEM, JEOL JSM 5600). Energy-dispersive spectroscopy (EDS) analyses were performed to obtain qualitative insights on the elemental composition of MoAlB and MBene nanostructures. Imaging was performed at an acceleration voltage of 10 kV, while the EDS used 20 kV. EDS measurements were performed at a working distance of 5 mm, while imaging maintained a working distance of 11.3 mm. Transmission electron microscopy (FEI TALOS F200S TEM 200 kV) were utilized to record TEM images and elemental mapping. X-ray photoelectron spectroscopy (XPS) were obtained using a Thermo Scientific K-Alpha. Nitrogen adsorption–desorption isotherms were measured cryogenic temperature using Micromeritics Tristar II surface area and porosimetry analyzer. According to the resulting isotherms of the MBene samples, the BET (Brunauer-Emmet-Teller) method was used for the calculation of surface area.

### 2.4 Preparation of 2D MBene Film Electrodes

In the process of electrode material preparation, first as-prepared 2D MoB MBenes (80 wt %) with a different etching ratio was dispersed in deionized water (DIW) and subjected to ultrasonic treatment for half an hour. Subsequently, polyvinylpyrrolidone (PVP) (20 wt %) was added to the solution to enhance the stability and cohesion of the mixture followed by stirring for 40 min at room temperature. The resulting solution was coated onto a nickel substrate using the drop-casting method. The coated electrodes were then dried in an oven at 60 °C for 15 min, promoting the evaporation of the solvent and the solidification of the electrode material.

### 2.5 Fabrication of Flexible Interdigitated Supercapacitor

**Figure 1:**
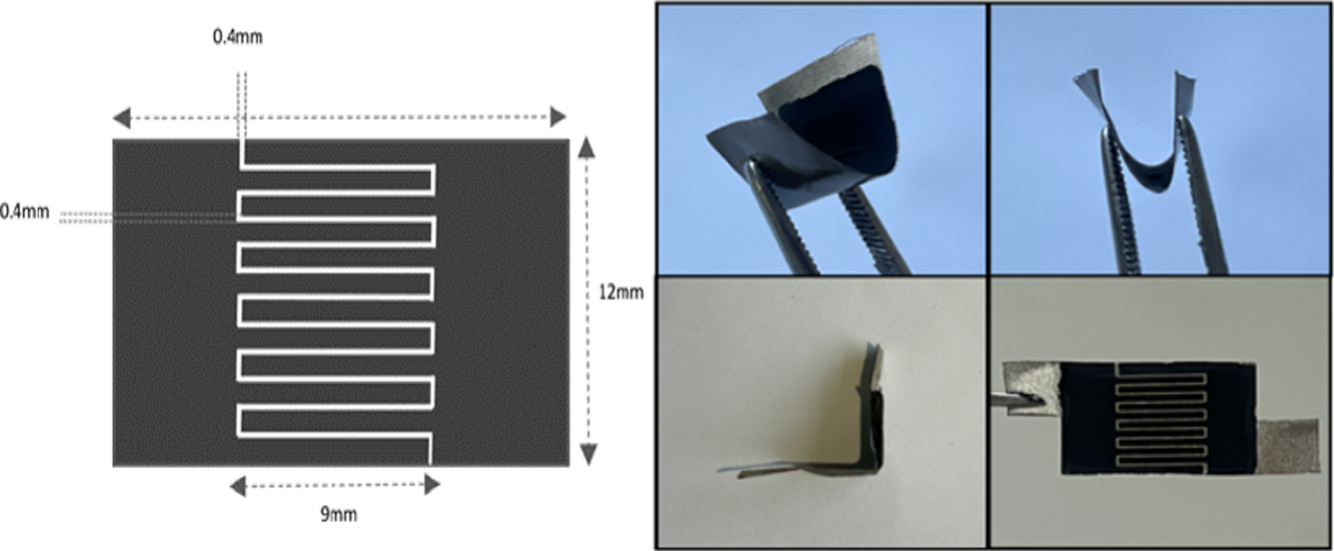
Illustration of electrode configuration of ISSC.

The resulting coating was developed on a flexible substrate patterned using a laser device to create an interdigitated pattern. A nickel band served a dual purpose as both the substrate and the current collector in the fabrication process. The serpentine arrangement of interconnects in the pattern was patterned using laser technology. The design was produced on a MoAl_1–*x*_B-coated nickel band, securely attached to the glasses to maintain the stability of the device throughout the measurement process. Figure 1 illustrates the dimensions and structure of the six fingers electrodes of the ISSC fabricated in this study. The spacing between the fingers was designed to be approximately 0.4 mm.

### 2.6 Electrochemical Measurements

The electrochemical experiments of symmetric ISSC devices were performed in a two-electrode setup. A nickel casing, functioning as a current collector, facilitates the connection of all components, including the anode, cathode, and PVA/H_2_SO_4_ gel electrolyte thereby establishing an integral system. The electrochemical studies for ISSC device were done using cyclic voltammetry (CV), galvanostatic charge–discharge (GCD), and electrochemical impedance spectroscopy (EIS). CV curves were obtained at different scan rates: 25, 50, 100, 150, and 200 mV/s in a suitable voltage window. GCD curves were obtained at different current values ranging from 0.4 to 2 mA. EIS was demonstrated in the Nyquist plot in the frequency range from 0.01 to 10^6^ Hz by applying a fixed potential of 20 mV.

#### 2.6.1 Theoretical Methods

Density functional theory (DFT) calculations were carried out using Quantum Espresso^23^ employing projected augmented wave (PAW) pseudopotentials^30,31^ and the Perdew–Burke–Ernzerhof (PBE) parametrization of the generalized gradient approximation (GGA) exchange-correlation functional.^32^ Brillouin zone sampling was performed on grids with k-point densities equivalent to a 30 × 30 × 1 grid for a 3 Å × 3 Å unit cell. Structural relaxation calculations included three orthorhombic phases: Mo_3_B_4_ in *Immm* and *Cmmm*, and MoB in *Cmcm*, along with three tetragonal phases: MoB in I41/amd, Mo_2_B_3_ in *P*4/*mbm*, and MoB_2_ in *I*4/*mcm*. Notably, the CmCm phase was experimentally observed in this study. Electronic band calculations were specifically conducted for the experimentally observed phase, CmCm. Plane-wave expansions were truncated at an energy cutoff of 60 Ry for wave functions and 480 Ry for charge densities. A Gaussian smearing scheme with a broadening of 0.01 Ry was applied. Additionally, a vacuum layer of 20 Å was introduced in the direction normal to the monolayer to minimize spurious interactions across the periodic boundary. The models depicted in the figures were visualized using VESTA software.^33^

## 3 Results and Discussion

**Figure 2:**
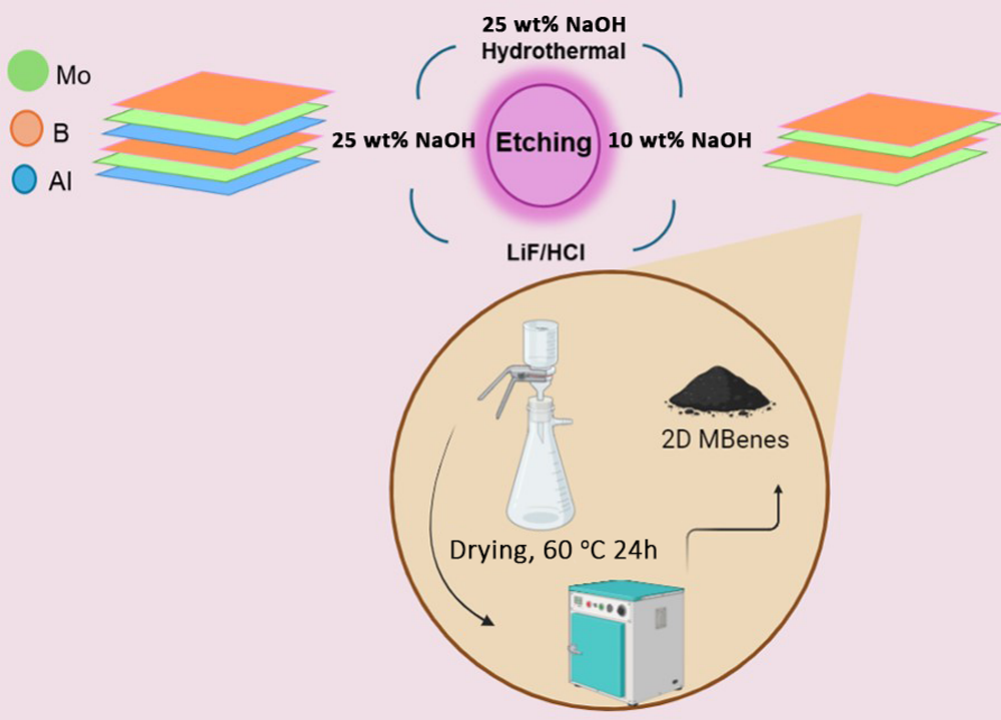
Schematic illustration of the synthesis procedure.

As in some MAX phases, it is known that the bond between the transition metal and aluminum is metallic and it is weaker than the covalent and ionic bonds between the transition metal and boron.^34^ As shown in Figure 2, in this work, we obtained 2D MoB nanostructures by exfoliating the Al layer of MoAlB using chemical etching (HCl, NaOH and LiF-HCl).^35^

**Figure 3:**
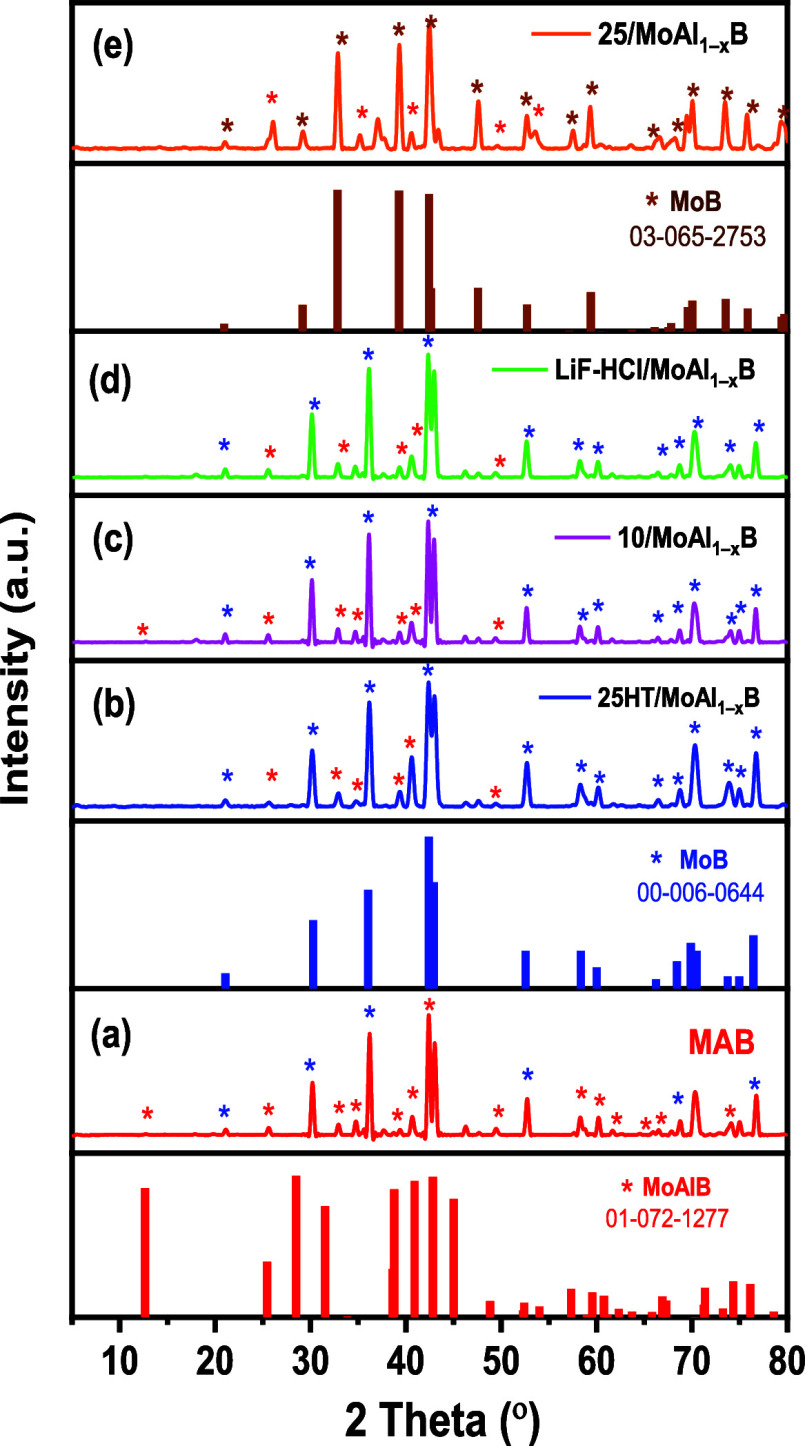
X-ray diffraction patterns of (a) MAB powder, (b) 25HT/MoAl_1–*x*_B, (c) 10/MoAl_1–*x*_B, (d) LiF-HCl/MoAl_1–*x*_B, and (e) 25/MoAl_1–*x*_B materials.

The PXRD analysis showed that the MoAlB was synthesized by the solid-state pressure less sintering method with a 2:1.5:2 (Mo: Al: B) molar ratio at 1500 °C for 10 h. Figure 3 depicts a comparison of the PXRD results of the MoAlB and different etching MBene samples (25HT/MoAl_1–*x*_B, 10/MoAl_1–*x*_B, LiF-HCl/MoAl_1–*x*_B, and 25/MoAl_1–*x*_B). The PXRD of the MoAlB powders synthesized in this study is shown in Figure 3a. When the PXRD diffraction pattern of the synthesized MAB phase compound is examined, it is observed to contain both orthorhombic MoAlB (*Cmcm* space group) and orthorhombic MoB (*Cmcm* space group) in its structure. It is seen that the MAB phase has the main peaks of MoAlB at 2θ angles of 12.64°, 25.45°, 28.48°, 31.52°, 33.86°, 38.59°, 38.77°, 40.92°, 42.83°, 45.00°, 48.83°, 52.29°, 52.41°, 54.00°, 57.32°, 58.96°, 59.55°, 60.76°, 61.15°, 62.33°, 63.71°, 65.82°, 66.90°, 67.32°, 70.30°, 71.24°, 71.36°, 73.24°, 74.32°, 76.11°, 78.57°, and 81.32°, assigned to the planes (020), (040), (110), (021), (130), (060), (041), (111), (150), (131), (061), (080), (151), (170), (200), (220), (002), (081), (022), (171), (240), (042), (0100), (112), (132), (260), (241), (062), (191), (152), (261), and (280), respectively, according to JCPDS data (JCPDS reference card number 01–072–1277). The main peaks of MoB are at 2θ angles of 21.08°, 30.27°, 36.04°, 42.40°, 42.93°, 52.55°, 58.357°, 60.02°, 66.22°, 68.42°, 69.87°, 70.48°, 73.72°, 74.95°, 76.44°, and 82.35°, assigned to the planes (020), (110), (021), (111), (130), (041), (200), (002), (060), (112), (151), (221), (061), (240), (042), and (241), respectively, according to JCPDS data (JCPDS reference card number 00–006–0644). After the etching procedure, as illustrated in Figure 3b–e, the appearing peaks of 25HT/MoAl_1–*x*_B, LiF-HCl/MoAl_1–*x*_B, and 10/MoAl_1–*x*_B are attributed to the orthorhombic MoB according to the JCPDS card.^36,37^ Also, in diffraction patterns of 25HT/MoAl_1–*x*_B, LiF-HCl/MoAl_1–*x*_B, and 10/MoAl_1–*x*_B, it was seen that the main peaks of MoAlB still exist, but it was observed that the peaks belonging to the orthorhombic MoB phase became more intense. As seen from Figure 3e, as a result of the NaOH/25% (25/MoAl_1–*x*_B) etching process, in diffraction patterns of 25/MoAl_1–*x*_B, it was seen that the main peaks of MoAlB still exist, it was observed that the intensity of some peaks decreased and the intensity of the peaks increased at 2θ angles 32.87°, 47.46°, 59.63°, 73.58°, 75.86° and 79.24°. This diffraction pattern is suggested to closely match the tetragonal MoB (I14/amd space group) (JCPDS reference card number: 03–065–2753) at 2θ angles of 20.94°, 29.17°, 32.85°, 39.28°, 42.43°,42.63° 47.55°, 52.70°, 57.13°, 59.38°, 60.48°, 63.69°, 66.09°, 67.51°, 67.82°, 68.86°, 69.55°, 70.02°, 73.54°, 75.82°, 79.39° and 79.67°, assigned to the planes (004), (101), (103), (105), (112), (008), (107), (116), (109), (200), (202), (204), (0012), (211), (1011), (206), (213), (1110), (215), (208), (217) and (1013).

**Figure 4:**
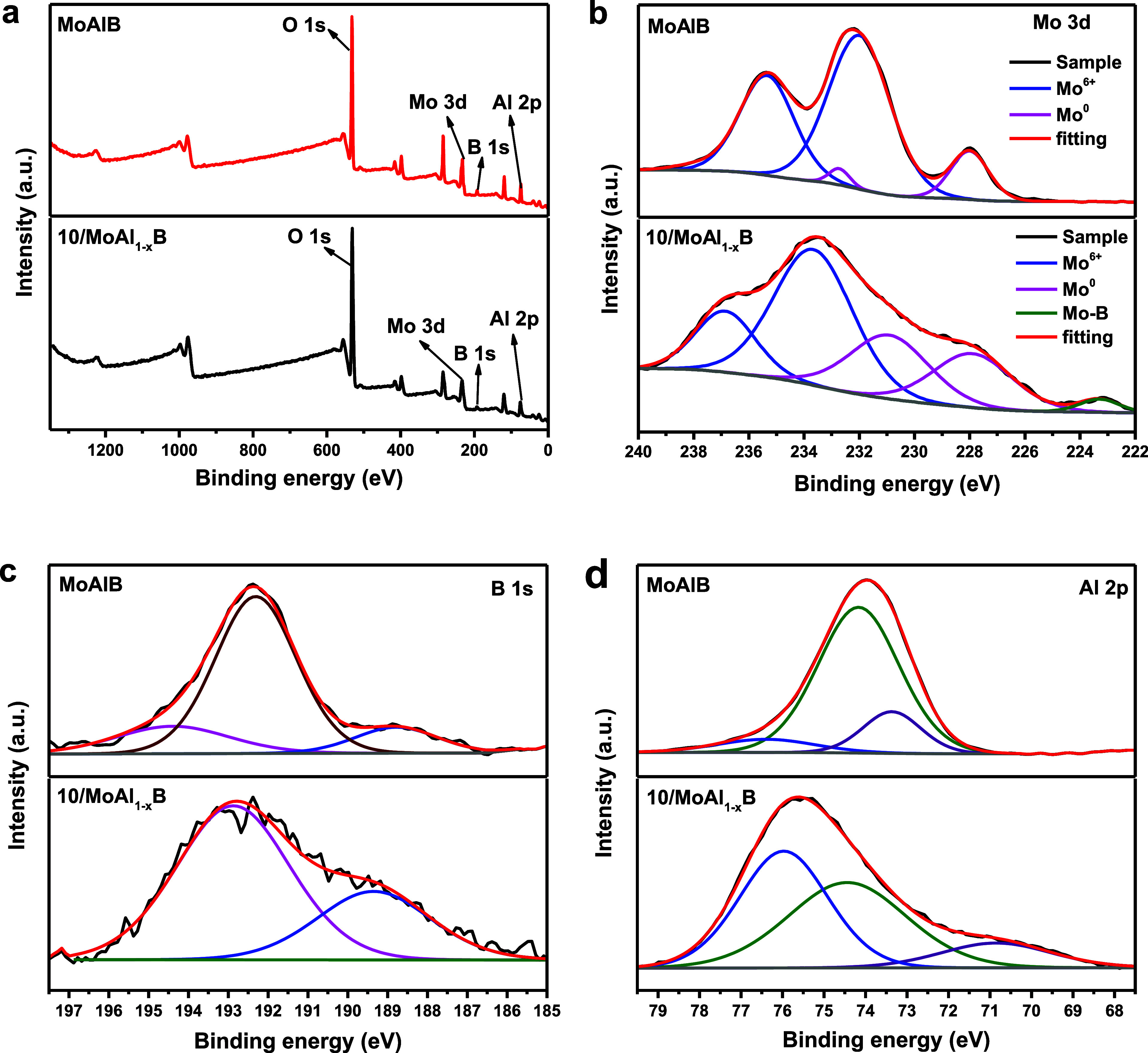
Comparison of XPS analysis (a) survey spectra, (b) Mo 3d, (c) B 1s, (d) Al 2p of MoAlB, and 10/MoAl_1–*x*_B samples.

The XPS analysis was treated to prove the changes in element composition and valence before and after the etching of the samples. Figure 4a and Table S1 represent a comparison of the survey spectra of various etched samples with the MoAlB phase. The elemental compositions of samples mainly consist of Mo 3d, O 1s, B 1s Al 2p. As shown in Mo 3d spectrum (Figure 4b), the Mo in MoAlB shows two pairs of peaks (Mo^0^, 3d_5/2_: 227.98 eV, 3d_3/2_: 232.71 eV, Mo^6+^, 3d_5/2_: 232.08 eV, 3d_3/2_: 235.48 eV) for metallic Mo and the oxidation state of Mo (VI). Also, a satellite peak was observed at a binding energy of 238.11 eV. When the spectrum of 10/MoAl_1–*x*_B is examined, MoB in the MBene phase shows one peak at a binding energy 223.29 eV and also, Mo presents two pair of peaks (Mo^0^, 3d_5/2_: 227.88 eV, 3d_3/2_: 230.88 eV, Mo^6+^, 3d_5/2_: 233.68 eV, 3d_3/2_: 236.98 eV) for metallic Mo and the oxidation state of Mo (VI). The binding energy of Mo (VI) 3d_5/2_ in MBene is higher than that in MoAlB, this is due to the oxygen atoms on the surface reducing the electron cloud density of molybdenum atoms.^20,27,38,39^ In B 1s spectra of MoAlB phase and MBene phase (10/MoAl_1–*x*_B) (Figure 4c), the small peak located on the 188.83 and 189.39 eV respectively may be ascribed to the typical Mo–B or B–B. The main peak at 192.38 eV and the small peak at 194.4 eV for the MoAlB phase, as well as the main peak at 192.86 eV for the MBene phase (10/MoAl_1–*x*_B), indicate the presence of B–O binding.^40,41^ As shown in Figure 4d, three individual fitted peaks of the Al 2p spectra at 73.36, 74.18, and 76.44 eV for the MoAlB phase, and at 70.77, 74.43, and 76.01 eV for the MBene phase (10/MoAl_1–*x*_B), are attributed to Al, MoAlB, and Al_2_O_3_, respectively. Additionally, the peaks in the Al 2p spectrum of the MBene sample appear to have shifted to lower binding energies, likely due to increased electronegativity as a result of the treatment with the etching solution. The quantitative Al etching performance is discussed in more detail in the EDS analysis, which is well-aligned with the XPS analysis.^42−44^

**Figure 5:**
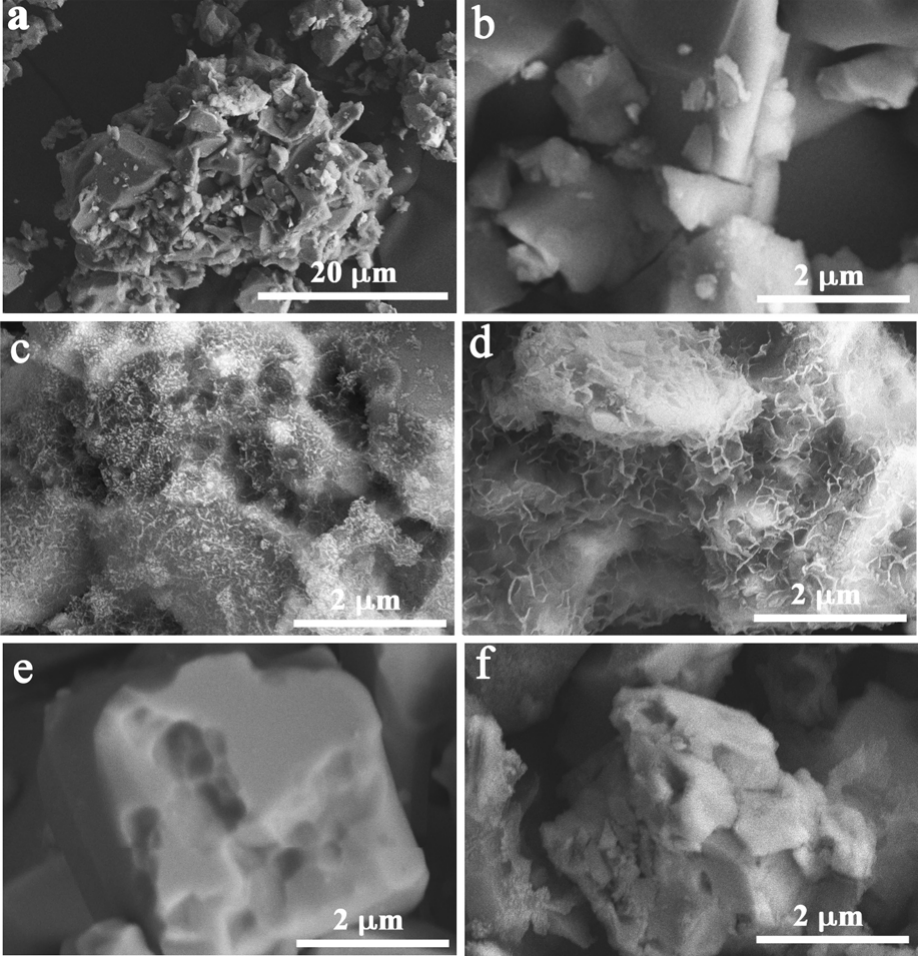
SEM images of (a, b) MoAlB at different magnifications, and (c) 25HT/MoAl_1–*x*_B, (d) 10/MoAl_1–*x*_B, (e) LiF-HCl/MoAl_1–*x*_B, and (f) 25/MoAl_1–*x*_B samples at 2 μm.

Figure 5a,b shows the morphological structure of the synthesized MoAlB powders. The image of MoAlB powders shows the existence of a very stacked structure. The SEM images of 25HT/MoAl_1–*x*_B, 10/MoAl_1–*x*_B, LiF-HCl/MoAl_1–*x*_B and 25/MoAl_1–*x*_B are illustrated in Figure 5c–f. Notably, the10/MoAl_1–*x*_B sample presents highly thin 2D morphology compared to the other sample owing to the most probably enhanced intercalation as well as the simultaneous delamination process that occurred by Na ions. It is worth noting that the LiF-HCl/MoAl_1–*x*_B sample presents stacked layers due to the weak delamination process and low ion size of Li compared to the Na ions.

The chemical composition of the 2D MoB nanostructures was examined using energy dispersive spectroscopy (EDS) presented in Table 2. The elemental composition of MBenes in Table 2 proves that Al was mostly removed from the MoAlB phase with the etching process as aligned with the XPS result. Based on Table 2, the maximum Al was removed from the MoAlB phase by applying LiF-HCl etching procedure followed by hydrothermal NaOH treatment which verify that hydrothermal NaOH and LiF-HCl are favorable for interlayer etching of Al.

**Table 2:** EDS Analysis Results of Samples

sample	Mo (wt %)	Al (wt %)	B (wt %)	O (wt %)	F (wt %)
MoAlB	32.8	43.8	13.94	9.4	
25HT/MoAl_1–*x*_B	70.60	2.98	18.20	8.19	
10/MoAl_1–*x*_B	52.00	9.74	26.32	11.93	
LiF-HCl/MoAl_1–*x*_B	43.304	3.844	39.84	4.52	8.48
25/MoAl_1–*x*_B	71.23	2.99	14.29	11.49	

**Figure 6:**
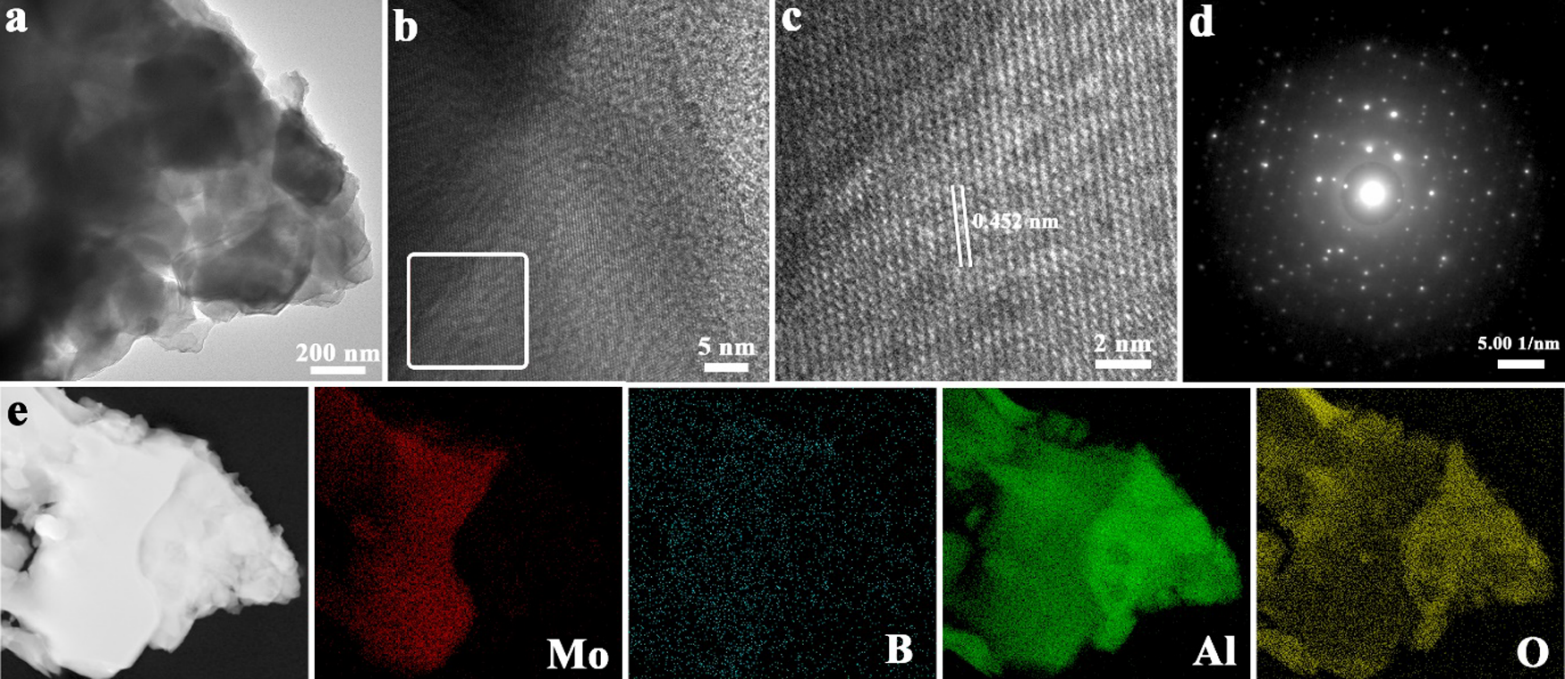
(a–c) HRTEM image, (d) SAED image, and (e) elemental mapping of 10/MoAl_1–*x*_B.

The high-resolution TEM (HRTEM) images of 2D MoB MBene are presented in Figure 6. The HRTEM image shows the high crystallinity of 2D MoB MBene with a lattice spacing of 0.452 nm, corresponding to the (112) plane. The corresponding elemental mapping images (Figure 6e) of 2D MoB MBene reveal a composition of uniformly distributed Mo, B, Al and O elements.

In Figure S1, N_2_ adsorption–desorption isotherm with hysteresis loops on MBene materials was observed, which was an important feature of ordered mesopores. The BET surface area of 10/MoAl_1–*x*_B was calculated to be 5 m^2^/g, respectively. Also, the average pore diameter and total pore volume of 10/MoAl_1–*x*_B material were calculated at 40.9 nm and 0.0184 cm^3^/g.

XRD, SEM and XPS analyses reveal the major changes when the same material is subjected to different chemical treatments for the same period of time. For example, although the 25HT/MoAl_1–*x*_B material was exposed to both 25% NaOH solution and a higher temperature hydrothermal treatment, the crystal structure remained the same as the other samples. However, although the 25/MoAl_1–*x*_B sample was obtained as a result of a reaction carried out in 25% NaOH solution and room conditions, the dominant crystalline phase in the MoAlB phase disappeared according to the diffraction pattern, and a new crystal phase was formed (tetragonal MoB). This may be a result of etching using 0.25 g less material than the others. In addition, it is seen that the morphological structures in the SEM analyses are very similar to each other despite the NaOH etching being carried out at different temperatures and different concentrations in the 10/MoAl_1–*x*_B and 25HT/MoAl_1–*x*_B samples. This conclusion was reached that high pressure and temperature increased Al removal.

### 3.1 Electrochemical Studies of MoAl_1–*x*_B Materials

**Figure 7:**
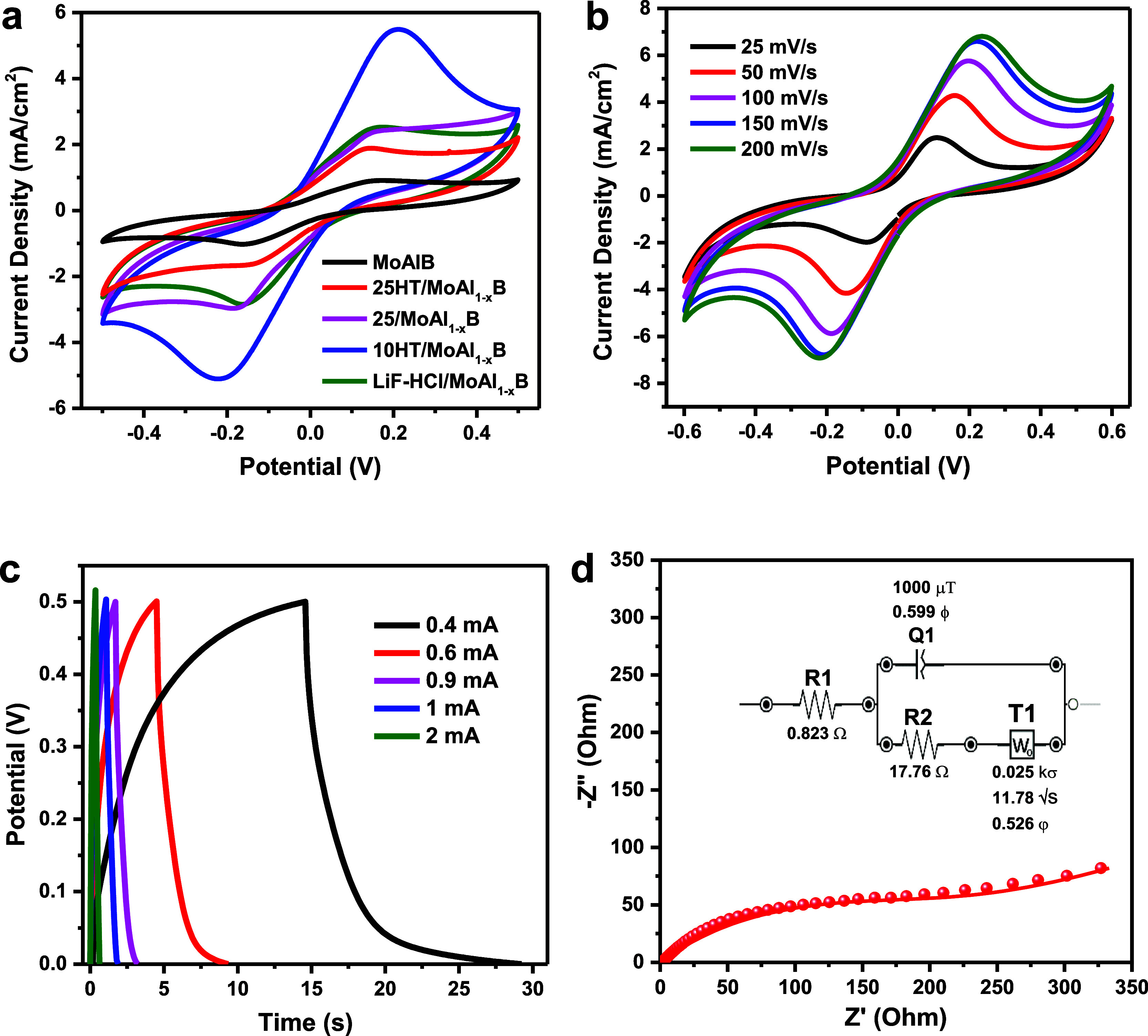
Electrochemical measurements for ISSCs in the 2-electrode setup (PVA/H_2_SO_4_ gel electrolyte) (a) CV curves specifically for ISSCs coated with MoAlB, 25HT/MoAl_1–*x*_B, 10/MoAl_1–*x*_B, LiF-HCl/MoAl_1–*x*_B and 25/MoAl_1–*x*_B at 100 mV/s and (b) CV curves at different scan rates, (c) GCD profiles at various current values, (d) Nyquist plots obtained via EIS of ISSCs coated with 10/MoAl_1–*x*_B.

The electrochemical behaviors of ISSCs in which MoAlB and different etching MoAl_1–*x*_B products were used as electrodes were examined by cyclic voltammetry method. In Figure 7a, the CV curves of ISSCs prepared using MoAlB and four different MoAl_1–*x*_B (25HT/MoAl_1–*x*_B, 10/MoAl_1–*x*_B, LiF-HCl/MoAl_1–*x*_B, and 25/MoAl_1–*x*_B) electrodes at 100 mV/s offer valuable insights into their electrochemical performances. All ISSCs have a nonrectangular CV curve indicating the presence of pseudocapacitive behavior. Especially the ISSC prepared using 10/MoAl_1–*x*_B sample has a higher enclosed area with the largest peak currents compared to the other electrodes and stands out with its good capacitance levels. Cyclic voltammetric responses of the 10/MoAl_1–*x*_B ISSC were generated at different scan rates ranging from 25 to 200 mV/s within a potential range of −0.5 to 0.5 V, as depicted in Figure 7b. The CV curves of ISSC coated with 10/MoAl_1–*x*_B electrode formed a reversible redox couple. A reversible redox couple is observed in the CV curves of ISSC coated with 10/MoAl_1–*x*_B electrode. These CV curves show nonrectangular shapes with apparent redox peaks. The pair of well-defined redox peaks indicates the significant faradic behavior of prepared electrode material. As the scan rate increases, the capacity decreases due to rapid reactions occurring at the electrolyte and electrode interface. Concomitantly, the anodic peaks shift toward the positive direction, while the cathodic peaks shift toward the negative direction, implying that relatively low resistance and rapid redox reactions occurring at the interface between the electrode and the electrolyte.

The GCD curves of ISSC coated with 10/MoAl_1–*x*_B electrode at different current values depicted in Figure 7c exhibit quasi-triangular shapes with nonlinearity and asymmetry at various current values, highlighting the significant influences of pseudocapacitance. At lower currents, it can be said that the electrolyte ions get more time to penetrate into the inner plane of the electrode material. Hence, both the inner and outer surfaces contribute to enhancing the electrochemical performance at lower currents, which leads to an increase in the specific capacitance of the material. At higher currents, the interaction of electrolyte ions occurs only with outer surfaces of the electrode material because of limited time access of the electrolyte ions. As a result, a decrease in the C_sp_ can be observed. Also, as seen in the figure, there is no obvious voltage drop at high charge–discharge speed. The capacitance values of ISSC coated with 10/MoAl_1–*x*_B electrode were calculated as 20.3, 10.6, 6.7, 3 and 2.3 mF/cm^2^ at current values of 0.4, 0.6, 0.9, 1 and 2 mA, respectively. These values are comparable to those of ISSCs prepared using electrode materials such as MXene,^45−51^ graphene,^52−54^ and boride.^55,56^ This remarkable performance of the 10/MoAl_1–*x*_B sample underscores the importance of its composition of the enhanced etching process, particularly in shaping its electrochemical behavior. The elevated capacitance of the 10/MoAl_1–*x*_B electrode promises exciting possibilities for advancing energy storage technologies.

The EIS measurement of the device was performed in an open voltage from 0.01 Hz to 1000 kHz frequencies as illustrated in Figure 7d. Notably, the X-intercept in the Nyquist plots at higher frequencies pointed to an internal resistance of 0.823 ohm. The presence of smaller-diameter semicircles at higher frequencies suggests a reduction in charge transfer resistance at the electrode–electrolyte interface. Furthermore, the low-frequency plot reveals the slop less than 45° indicates a faster Warburg element, this observation suggests that diffusion processes at the electrode interface operate more efficiently in our electrochemical system.

**Figure 8:**
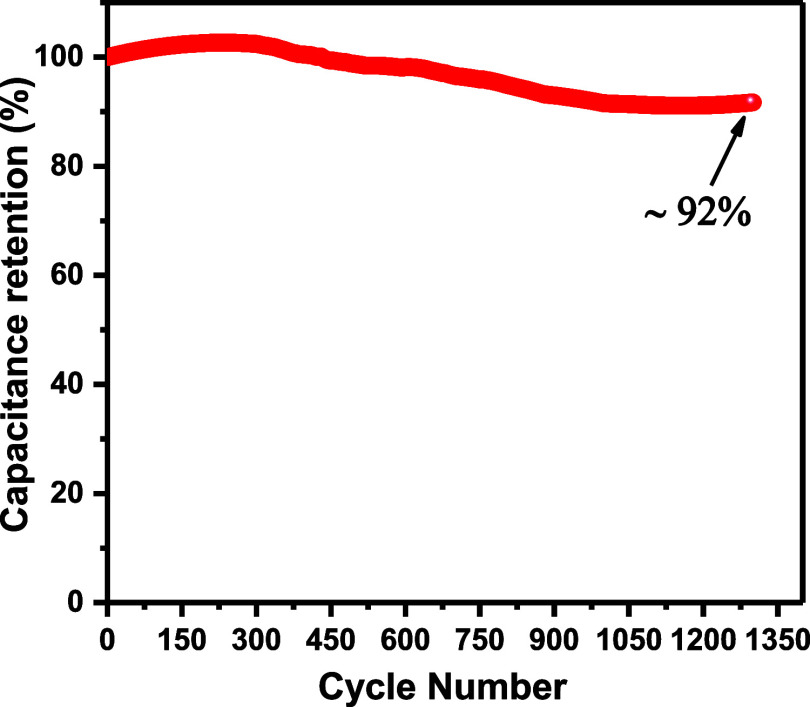
Illustration of cycling stability of the ISSC coated with 10/MoAl_1–*x*_B electrode throughout 1300 cycles.

A cycling stability test at a scan rate of 100 mV/s was conducted to assess the behavior of the of the ISSC coated with the optimized 10/MoAl_1–*x*_B electrode. Figure 8 visually presents the results showcasing the capacitance retention throughout these 1300 cycles. The ISSC exhibits remarkable cycling stability, maintaining approximately 92% of its original capacitance in PVA/H_2_SO_4_ gel electrolyte.

**Figure 9:**
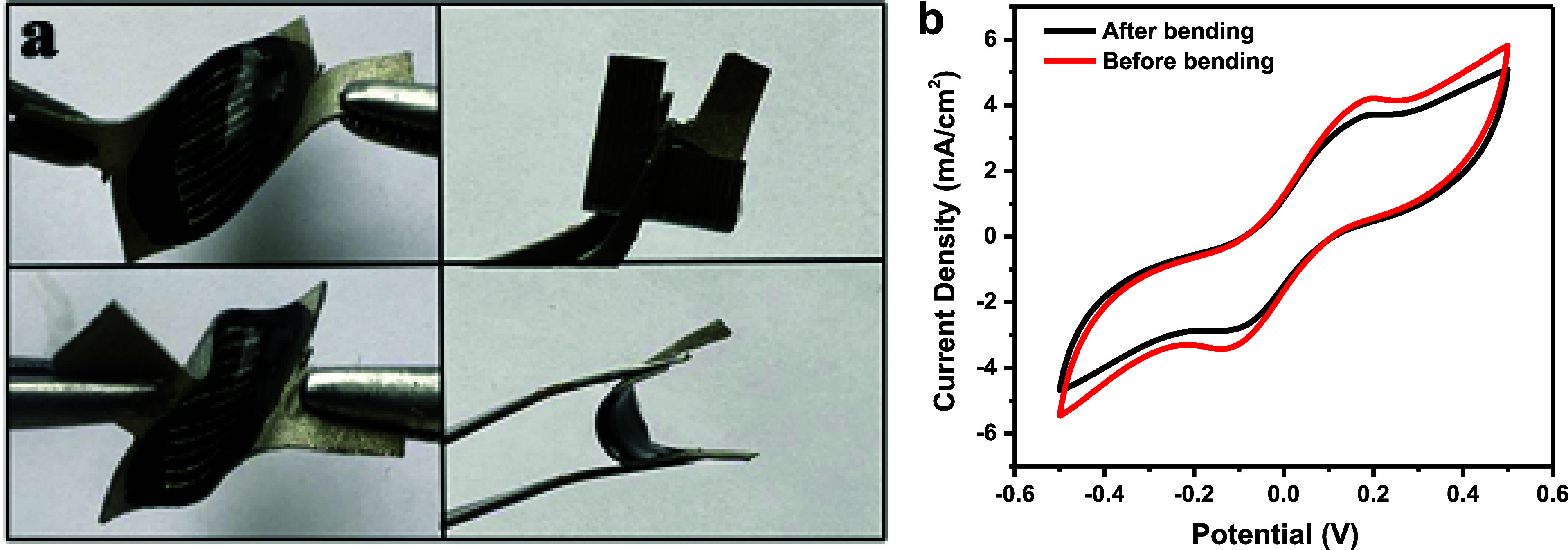
(a) Illustrates the electrode flexibility under bending in different angles and sides, and (b) the CV performance of the electrode before and after bending.

A film electrode, possessing remarkable flexibility and a thickness as minimal as approximately 130 μm, has been successfully manufactured. This electrode possesses the unique ability to be contorted into various shapes, as visually depicted in Figure 9a. Upon revisiting the electrode’s performance through CV curve analysis postbending, little changes emerged. Figure 9b illustrates the CV curve of the ISSC before and after bending on different sides and angles, as depicted there was a minor decrease in the area of the curve of electrodes that experienced slight alterations. Nevertheless, the electrode demonstrated resilience, maintaining functionality despite the applied bending condition. These intricate observations yield valuable discernment into the electrodes reaction to mechanical distortion, culminating in a comprehensive comprehension of its potential implementations in the realm of adaptable electronics and associated domains.

### 3.2 Structures of Predicted 2D Molybdenum Boride

**Figure 10:**
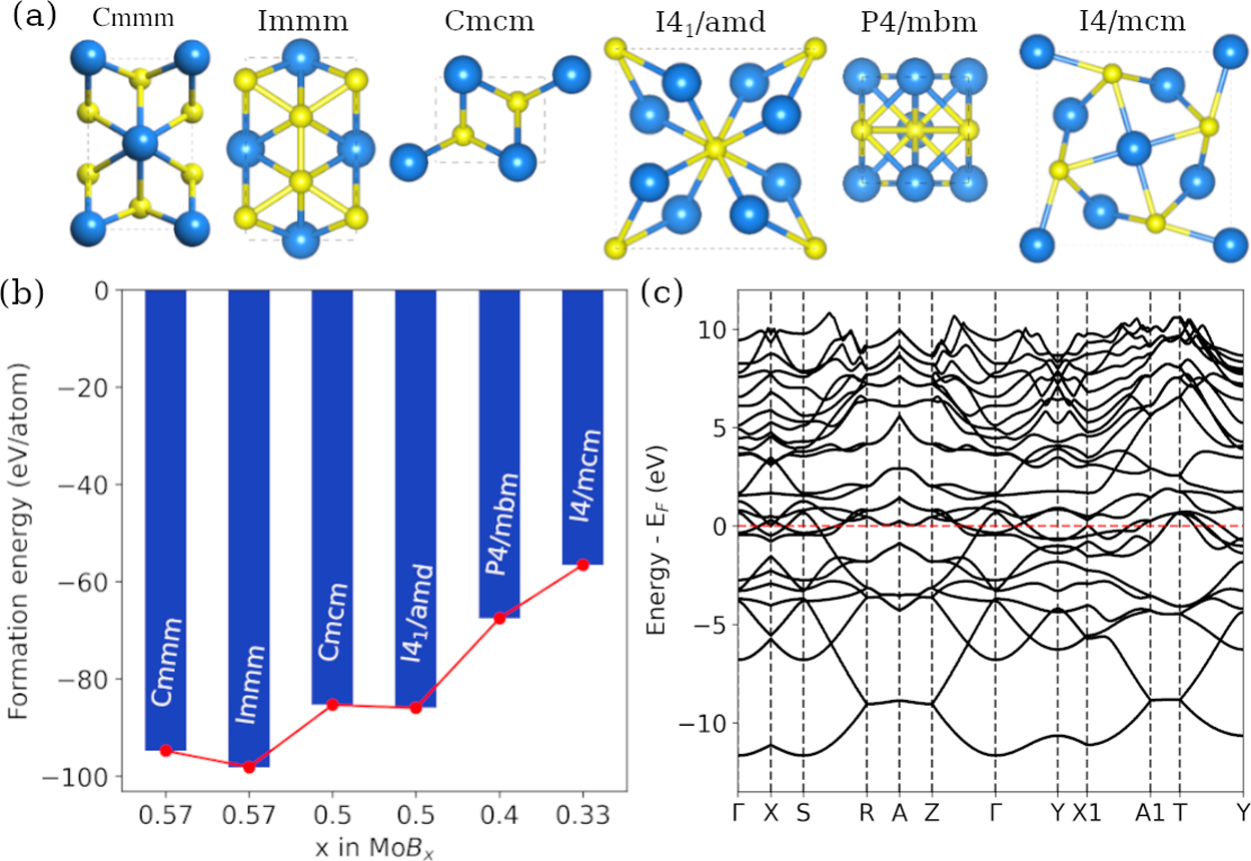
(a) DFT optimized configurations of six MBene monolayers in orthorhombic (Mo_3_B_4_ in *Immm* and *Cmmm*, and MoB in *Cmcm*) and tetragonal phases (MoB in I41/amd, Mo_2_B_3_ in *P*4/*mbm*, and MoB_2_ in *I*4/*mcm*). (b) Formation energy per atom (in eV) of MoBx as a function of the B concentration. (c) Electronic band structure of MoB in the *Cmcm* phase.

Figure 10a presents the optimized configurations of orthorhombic and tetragonal phases of MBene monolayers. Our analysis in Figure 10b indicates that Mo_3_B_4_ in the *Immm* phase exhibits the highest thermodynamic stability, followed by Mo_3_B_4_ in *Cmmm* and MoB in *Cmcm*. This suggests that although the CmCm phase is observed in experiments, both *Immm* and *Cmcm* phases are anticipated to be experimentally accessible. Additionally, there is a slight disparity in the formation energies between the orthorhombic *Cmmm* and tetragonal *I*4_1_/*amd* phases, implying that both phases may coexist in MBene from a thermodynamic perspective. Another notable finding from the calculations is that the thermodynamic stability of an MBene monolayer increases with the increase in the B-concentration, indicating a thermodynamic preference for B-rich conditions during the monolayer formation. MBenes in the *Cmcm* phase exhibit excellent metallic behavior, as evidenced by multiple bands crossing the Fermi energy in Figure 10c.

## 4 Conclusions

In conclusion, this study successfully demonstrated the synthesis of 2D MoB MBenes from MoAlB phases via chemical exfoliation using NaOH and LiF-HCl etching solutions. The selective extraction of interlayer aluminum played a critical role in forming 2D MoB MBene nanostructures with enhanced physical and electrochemical properties. Among the synthesized materials, the ISSC device coated with 10/MoAl_1–*x*_B electrode exhibited exceptional electrochemical performance, with a high areal capacitance of 20.3 mF/cm^2^ and a remarkable 92% retention of capacitance after 1300 cycles, showcasing its potential for long-term stability in energy storage applications. Structural characterization through PXRD, SEM, and TEM confirmed the crystallinity, phase purity, and sheet-like morphology, while BET surface area analysis provided insights into the porous nature crucial for electrolyte interaction. XPS analysis further verified the successful removal of aluminum and the formation of pure MoB MBene, confirming the elemental composition and oxidation states necessary for enhanced redox activity. Furthermore, the development of a Density Functional Theory (DFT) model aligned well with experimental results, offering insights into the thermodynamic stability, conductivity, and structural integrity of the synthesized MoB MBene. This theoretical approach, combined with experimental validation, underscores the robustness and reliability of the material’s performance. The promising results suggest that these 2D MoB MBene nanostructures, particularly the 10/MoAl_1–*x*_B sample, can serve as effective all-solid-state flexible interdigitated electrodes in supercapacitor applications, paving the way for their use in next-generation energy storage devices. The findings of this study contribute significantly to the growing field of 2D transition metal borides, offering a new material platform with electrochemical performance and flexibility, for advancing supercapacitor technology.

## Supporting Information Available

XPS peak binding energy (eV) results for elements and surface area analysis of 10/MoAl_1–*x*_B (PDF)
