# Supplementary material for: Synthesis, Characterization, and Theoretical Modeling of 2D Molybdenum Boride (MBene) for All Solid-State Flexible Interdigitated Supercapacitor Application
Source: ACS Omega. 2025 Feb 19;10(8):8202–12. doi: 10.1021/acsomega.4c09727 (PMC11886652; doi:10.1021/acsomega.4c09727)
Supplement: Supplementary file 1 — ao4c09727_si_001.pdf [file ao4c09727_si_001.pdf]

Synthesis, Characterization, and Theoretical modeling of 2D Molybdenum Boride (MBene) for all-solid-state flexible interdigitated supercapacitor application

Parya Aghamohammadi[a], Fatma Karakaya Mert[a], Eda Taga Akgul[a], Nahid Aghabalapoor Keshtiban[b], Osman Cem Altıncı[a], Ali Gelir[b], Cem Sanga[b], Nadire Nayir[b], Hamide Aydın[c] and Muslum Demir[c,d*]

[a] Osmaniye Korkut Ata University, Faculty of Engineering and Natural Sciences, Department of Chemical Engineering, 80000, Osmaniye, Türkiye

[b] Istanbul Technical University, Department of Physics Engineering, 34469, Istanbul, Türkiye

[c] TUBITAK Marmara Research Center, Material Institute, 41470, Gebze, Türkiye

[d] Bogazici University, Department of Chemical Engineering, 34342, Istanbul, Türkiye

Table S1: XPS peak binding energy (eV) results for elements

	MAB		25HT/$MoAl_{1-x}B$		10/$MoAl_{1-x}B$		LiF-HCl/$MoAl_{1-x}B$		25/$MoAl_{1-x}B$	
	Peak BE (eV)	Atomic (%)	Peak BE (eV)	Atomi c (%)	Peak BE (eV)	Atomi c (%)	Peak BE (eV)	Atomic (%)	Peak BE (eV)	Atomic (%)
**Al 2p**	75.19	10.37	75.02	6.37	–	–	75.65	12.55	75.06	7.95
**Si 2p**	–	–	–		–	–	–	–	103.89	7.74
**S 2p**	169.82	3.63	169.92	3.12	–	–	169.58	3.32	–	–
**B 1s**	193.16	2.24	192.08	7.44	–	–	–	–	193.25	4.71
**Mo 3d**	232.79	1.24	232.44	2.42	236.15	2.44	232.77	0.72	233.25	4.47
**C 1s**	285.27	24.35	285.16	38.36	288.04	24.39	285.2	28.55	285.15	24.67
**N 1s**	–	–	–	–	401.42	18.02	–	–	–	–
**O 1s**	532.51	55.77	532.14	42.29	535.12	55.15	532.2	50.85	532.31	42.28
**F 1s**	–	–	–	–	–	–	686.13	4	689.94	1.92
**Na 1s**	1071.8	2.39	–	–	–	–	–	–	–	–

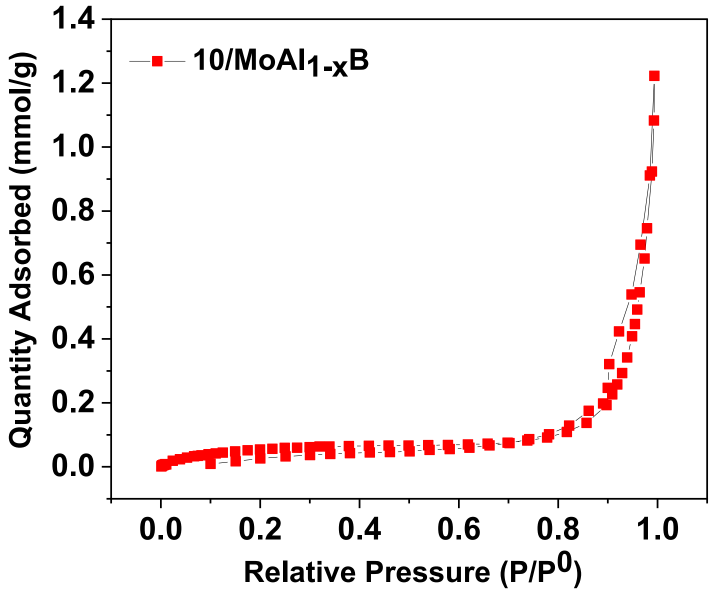

Fig S1. Surface area analysis of 10/$MoAl_{1-x}B$
